# Transitioning to adult care in youth-onset diabetes: a scoping review of socio-ecological factors in youth-onset type 2 diabetes compared to type 1 diabetes

**DOI:** 10.1186/s12889-025-22956-1

**Published:** 2025-05-15

**Authors:** Assumpta O. Ude, Sydney A. Dixon, Sophia B. Glaros, Sue-Ann Arboine, Nancy L. Terry, Tomás Cabeza De Baca, Stephanie T. Chung

**Affiliations:** 1 Clinical Center Nursing Department, National Institutes of Health, Bethesda, MD USA; 2 Section on Pediatric Diabetes, Obesity, and Metabolism, National Institute of Diabetes and Digestive and Kidney Diseases, National Institutes of Health, 10 Center Drive, BLD-10 CRC, RM 5-5942, Bethesda, MD 20814 USA; 3 National Institutes of Health Library, National Institutes of Health, Bethesda, MD USA; 4 Obesity and Diabetes Clinical Research Section, Phoenix Epidemiology and Clinical Research Branch, National Institute of Diabetes and Digestive and Kidney Diseases, Phoenix, AZ USA

**Keywords:** Youth-onset type 2 diabetes, Type 1 diabetes, Socio-ecological factors, Psychological factors, Transition care, Scoping review, Barriers, Facilitators, Health cultural factors

## Abstract

**Background:**

Multiple socio-cultural and personal factors influence the transition from pediatric to adult-centered diabetes care in youth-onset type 1 diabetes (Y-T1D), but few data exist in youth-onset type 2 diabetes (Y-T2D). We determined the scope of the literature on socio-ecological factors associated with transitioning to adult care in Y-T2D compared with Y- T1D to identify facilitators, barriers, and knowledge gaps in Y-T2D.

**Method:**

We conducted a global scoping review using the Systematic Reviews and Meta-Analyses for scoping reviews (PRISMA-ScR) guidelines. Eligible articles were peer-reviewed experimental and quasi-experimental articles published between January 1990 and September 2022, with no language restrictions.

**Results:**

In 104 articles that reported on transitioning to adult care, 88% were in Y-T1D, 6% compared Y-T1D and Y-T2D, 2% reported in Y-T2D only, and 4% reported on youth-onset diabetes diagnosis. The proportion of articles that reported on socio-ecological domains were similar in Y-T1D compared to articles that included Y-T2D. Identified challenges associated with the transitioning period in Y-T2D were societal (structural bias, poverty, inadequate social support), health cultural (limited access to adult health-care providers), psychological (feelings of anxiety and fear of transition), and behavioral (difficulty with medication adherence). The transition period was associated with worsening glycemic control in both groups.

**Conclusions:**

Y-T2D face multiple challenges across socio-ecological domains during the transition to adult-centered diabetes care. However, only 8% of studies on transitioning factors included Y-T2D and additional research is needed to develop dynamic and robust transition programs in Y-T2D.

**Scoping review registration:**

Protocol was registered with Open Science Framework, April 4, 2022. https://osf.io/k2pwc.

**Supplementary Information:**

The online version contains supplementary material available at 10.1186/s12889-025-22956-1.

## Introduction

Youth-onset type 1 (Y-T1D) and youth-onset type 2 diabetes (Y-T2D) are among the most common chronic conditions of childhood. The annual incidence of Y-T1D is 22 per 100,000 and 18 per 100,000 in Y-T2D with rates increasing worldwide [1–3]. Contemporary treatment regimens for both types of youth-onset diabetes—including novel pharmacologic agents and device-assisted technologies—dramatically optimized diabetes care and markedly improved quality of life [4]. However, for many youths, there are persistent challenges in accessing these treatments. Further, diabetes-related distress and complications are ever-present realities that contribute to disease-related burdens during adolescence [5]. Socio-economic (for example, food and housing insecurity) and personal challenges (including depression and mood conditions) related to diabetes care often begin during childhood and may peak during adolescence [6]. Moreover, diabetes burdens are magnified by the uncertainty and anxiety that come with emerging into adulthood. The normative emerging adulthood period—which may begin as early as 13 years of age and continue through 25 years of age —marks a major shift to autonomy in thought, self-identification, and behaviors [7]. This period is critical for reinforcing diabetes-related self-management strategies aimed at optimizing long-term health. Structured clinical programs are essential for supporting adolescents during this developmental period to promote autonomy, optimize health outcomes and healthcare utilization, and promote positive health system experiences [8, 9]. However, the transition period from pediatric to adult care diabetes services has been strongly linked to poor outcomes and worsening diabetes control in Y-T1D and there are scarce data in Y-T2D [10, 11].

Factors that promote or impede the transitioning of diabetes care in Y-T1D are well described and include socio-cultural, psychological, and structural ecological factors (such as access to health care, financial burdens, and gender biases) [11]. Psychological factors, including mood disorders and community relationships are established challenges that influence transitioning of care in Y-T1D [12]. Emerging Y-T1D must balance increasing financial and social independence with burdensome diabetes self-management tasks including carbohydrate counting, insulin pump or injection management, and multiple daily blood glucose monitoring. Successful transition programs in Y-T1D employ coordinated, purposeful, and planned multistep processes to facilitate uninterrupted care delivery to optimize glycemic control [13].

The barriers and facilitators for successful transitioning to adult care in Y-T2D are not as well-established or understood. Factors promoting healthy transitioning in Y-T2D may be distinct from those reported in Y-T1D because the social, personal, and diabetes care obstacles may differ between the two conditions. In contrast to Y-T1D, self-management in Y-T2D may include an array of treatment strategies ranging from diet and lifestyle interventions to daily oral medications and/ or weekly incretin analogues to frequent insulin injections and continuous glucose monitoring. Moreover, novel anti-diabetes agents, such as sodium glucose transporter-2 inhibitors and once weekly glucagon-like 1 receptor agonists, increase the suite and choice of diabetes agents available to Y-T2D [14]. Adherence to non-insulin medications may be challenging. Contemporary medicines may require dose escalation over weeks to months and understanding which medications are covered by health insurance can be daunting. Navigating prescription drug and device management and social determinants of health could pose additional barriers during transition for Y-T2D [15], but whether these factors have been systematically evaluated remains unanswered.

Therefore, to comprehensively compare factors associated with transitioning care in Y-T1D and Y-T2D, we used the socio-ecological framework a conceptual method of describing the relationship between individual, community, and societal level influences [16]. This model facilitates a broad map of the literature to identify known mediators and intervention areas for future research [17–19]. Our objectives were to determine the scope of the literature on (1) factors within the socio-ecological framework (societal, community/ relationship, individual/ biological) associated with transitioning to adult care in Y-T2D compared with Y- T1D, (2) facilitators and barriers to transitioning to adult care in Y-T2D, and (3) to identify knowledge gaps related to transitioning to adult care in Y-T2D.

## Materials and methods

### Design and search strategy

A scoping review was conducted to map the state of the literature on transitioning care in youth-onset diabetes and to identify the main sources and types of evidence available. The detailed protocol, including search terms that adhered to the Preferred Reporting Items for Systematic Reviews and Meta-Analyses for scoping reviews (PRISMA-ScR) and JBI methodological framework and the search strategy was published [17]. The proposed scoping review protocol was registered with Open Science Framework on 4 April 2022 (OSF Registries| Socio-ecological Factors in Transitioning from Youth to Adult Diabetes Care: A Scoping Review Protocol). In summary, we completed a systematic search of the Embase, PubMed, and Cumulative Index of Nursing and Allied Health (CINAHL), PsycINFO and Scopus databases of experimental and quasi-experimental studies published. As described in the protocol, we used the following main terms: diabetes, youth and young adults, sociologic, psychological, and socioecological factors [17].

Eligibility criteria for article selection included a main outcome related to transitioning from youth to adult diabetes care and a publication period between from January 1,1990 to September 30, 2022. Initial abstract screening inclusion criteria were children/ young adults age 0–25 years with a diagnosis of Y-T2D and Y-T1D. After study screening, the inclusion criteria for the protocol was amended to include youth-onset diabetes (either Y-T1D, Y-T2D, or unspecified) and to remove the age criteria. These changes were made because four studies referenced youth-onset diabetes but did not specifically define Y-T1D or Y-T2D. In addition, several studies did not report age or included both young and older adults. These changes were justified because of the broad objectives for this scoping review and the goal of capturing all studies related to transitioning to adult care, regardless of age. Peer-reviewed, original experimental and quasi-experimental study designs were included, and there was no imposed race, ethnicity, country, or language restrictions to facilitate a broad global search [17]. Non-English printed articles were translated to English by a certified translator (NIH Library) for review. We included original articles only. Systematic or narrative reviews, commentaries, experiential articles, abstracts, anecdotal reports, and unpublished articles were excluded from this scoping review [17].

### Data extraction and data synthesis

The data extraction plan and outcomes were developed a priori [17]. Extracted elements from each study included: study design, year of publication, country of study, characteristics of the sample, and variables studied. Data items associated with transitioning from pediatric to adult diabetes care were categorized by socio-ecological level: (1) societal/ social determinants of health (structural bias, food insecurity, poverty, medical infrastructure and access to multidisciplinary care), (2) relationship/ community factors (psychological and behavioral factors including resiliency, anxiety, depression, diabetes management and medication adherence support, attitudes towards healthcare, health cultural attitudes, social support systems, or adverse childhood events) and (3) individual characteristics (biological sex, age, race/ethnicity, hemoglobin A1c (HbA1c), self-management behaviors). Citations were imported into Covidence (Melbourne, Australia) for title, abstract and full text screening. This was a descriptive scoping review, and no formal statistical hypothesis testing was performed. Three review authors (SD, AU, and TC) independently conducted the data extraction from each paper. Any discrepancies or uncertainties were resolved through discussion with the lead investigator STC after piloting the data charting process. Data from individual studies were extracted and collated in the Research Electronic Data Capture (REDCap) database. Studies were grouped by population: Y-T1D, Y-T2D, and Both/ Unspecified. Descriptive statistics were reported percentages, unless otherwise stated. Statistical analyses were performed in STATA (version 18.0, Stata Corp, College Station, TX, USA).

## Results

**Fig. 1:**
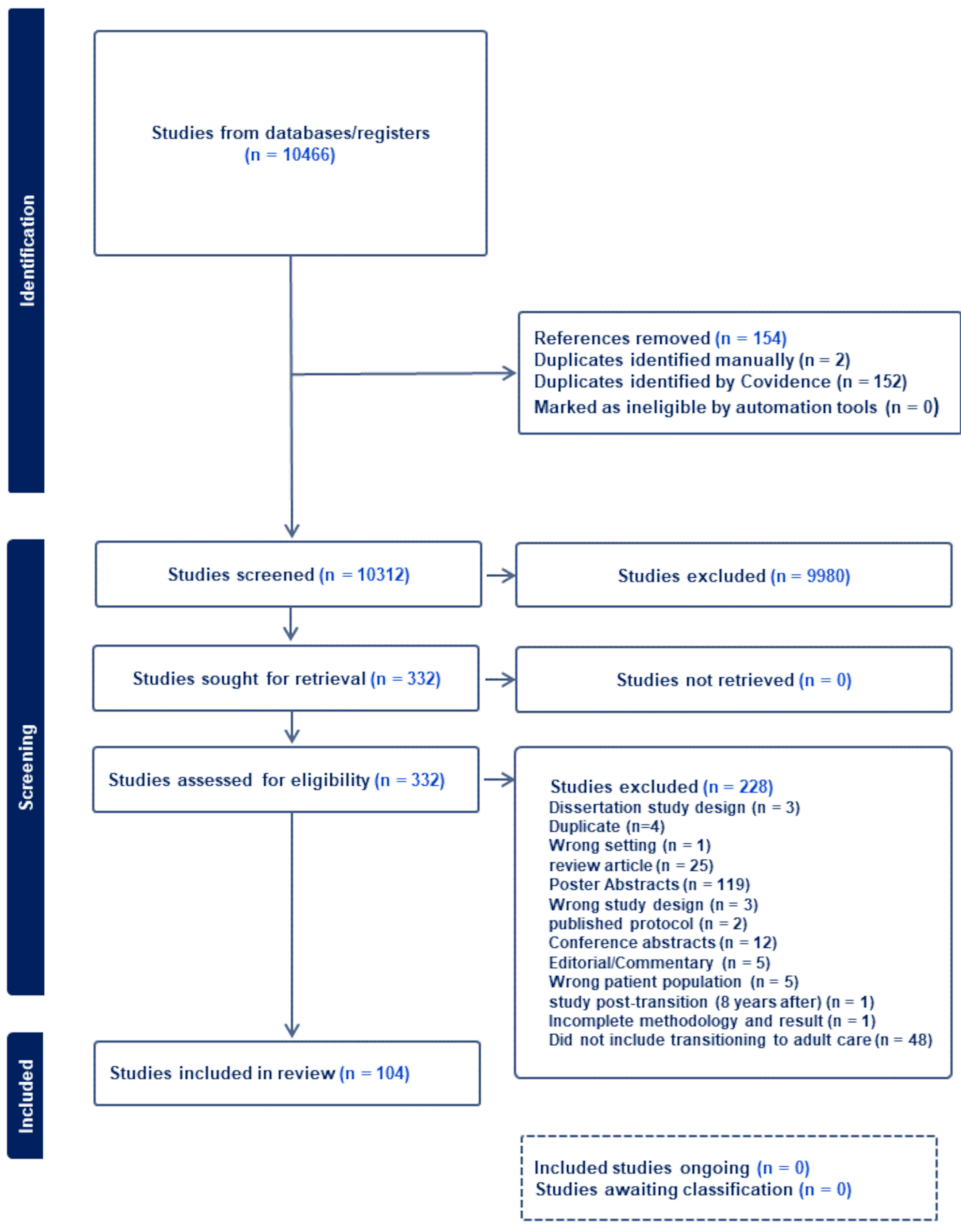
PRISMA-ScR flow diagram

**Fig. 2:**
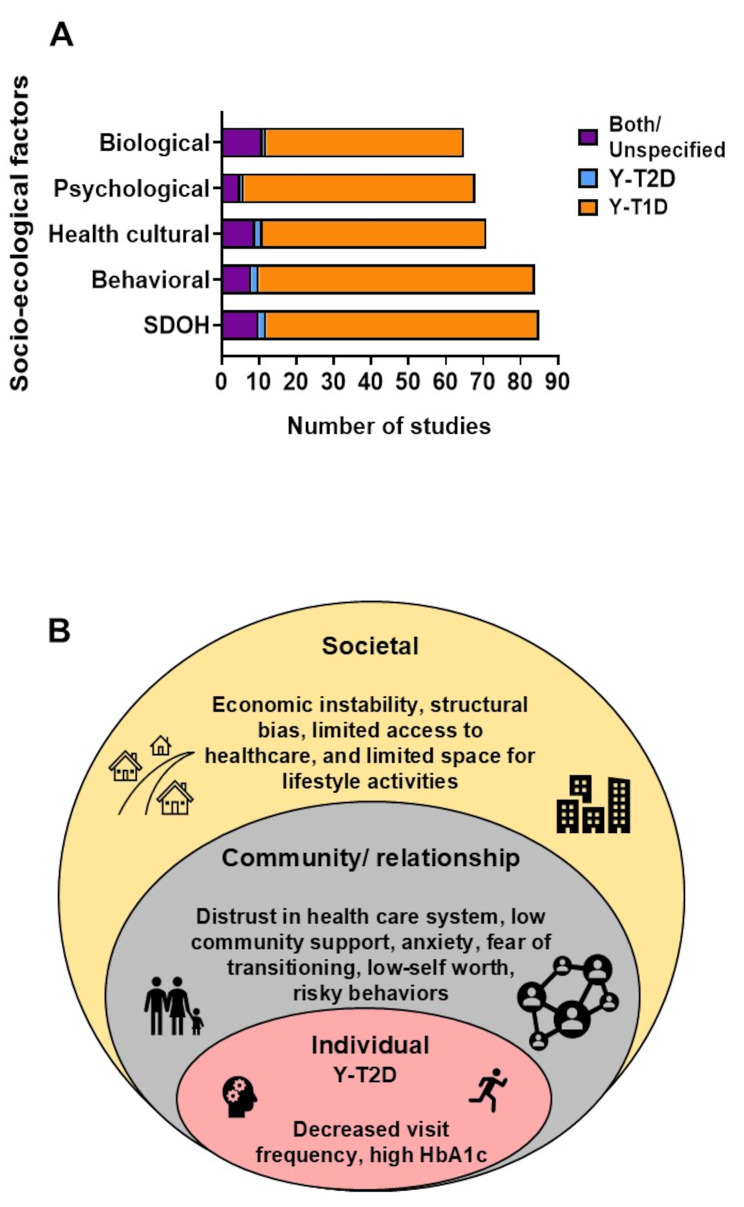
Socio-ecological Factors reported in studies of transitioning to adult diabetes care. (**A**) Bar graph showing percent of studies that reported social determinants of health (SDOH), health cultural factors, psychological factors, behavioral factors, and biological factors among all by population, Both/ Unspecified youth diabetes (purple), youth-onset type 2 diabetes (blue), and youth-onset type 1 diabetes (orange). (**B**) Reported barriers to transitioning to adult care conceptualized within the socio-ecological model. The model highlights the interrelationship between each level (circle) [91, 94–96, 98, 100]

Of the 10,466 articles identified, 332 articles were assessed for eligibility (Fig. 1). Of the 332 articles assessed for eligibility, 25 articles were reviews: two were systemic reviews, and 23 were narrative reviews. Although four of the narrative reviews mentioned Y-T2D in addition to Y-T1D, none of the reviews distinguished between factors influencing transitioning in Y-T1D and Y-T2D [13, 20–22]. One hundred and four articles were included in the scoping review, of which three were translated to English [23–25]. Only two studies were published before 2000 and the majority of studies that included Y-T2D were published after 2010. Table 1 shows the study design and population demographics of the 104 included studies. Most articles published (88%, *n* = 91) reported on transitioning of care in Y-T1D, 2% (*n* = 2) investigated transitioning in a Y-T2D cohort only, 6% (*n* = 7) compared Y-T1D and Y-T2D populations, and 4% (*n* = 4) did not specify the diagnosis type (Fig. 2; Table 2). Two-thirds of articles were conducted in a healthcare setting. Table 1 illustrates the study design and demographics grouped by population; 63% percent of studies reported on participants in North America with 52% percent in the United States of America (Table 1, Supplemental Table 1). Many participants were female and white ethnicity (Table 1), but only 43% of studies reported demographic data on race/ethnicity. 50% of studies used quantitative study design. In Y-T1D, there were: two randomized controlled, two non-randomized controlled, ten cohort, seventeen cross-sectional, fifteen longitudinal, thiry-seven qualitative, and eight mixed methods design. In studies that included Y-T2D/ Both: there were no randomized trials, two cohort, one cross-sectional, three longitudinal, three qualitative, and two mixed methods design.

**Table 1:** Population characteristics and socio-ecological factors reported in studies examining transitioning of diabetes care in youth with type 1 (Y-T1D) and type 2 (Y-T2D) diabetes

	All	Y-T1D	Y-T2D	Both/Unspecified
**Number of studies (N)**	104	91	2	11
**Study design.**				
- Qualitative	41 (39)	37 (41)	1 (50)	3 (27)
- Quantitative	51 (49)	43 (47)	1 (50)	7 (63)
- Mixed	12 (12)	11 (12)	0 (0)	1 (9)
**Study context.**				
- Community	26 (25)	22 (24)	1 (50)	3 (27)
- Healthcare	70 (67)	61 (67)	1 (50)	8 (72)
- Other	8 (8)	8 (9)	0 (0)	0 (0)
**Articles in English**	104 (97)	88 (97)	2 (100)	11 (100)
**Study Continent**				
- North America	65 (63)	55 (60)	1 (50)	9 (82)
- Europe	24 (23)	24 (26)	0 (0)	0 (0)
- Asia	5 (5)	4 (5)	0 (0)	1 (9)
- Africa	1 (1)	1 (1)	0 (0)	0 (0)
- Australia	9 (8)	7 (8)	1 (50)	1 (9)
**Study participant demographics**				
Number of studies reporting white race	47 (45)	41 (45)	1 (50)	5 (45)
Percent of white race in studies that reported race/ethnicity (%)	65	69	25	42
Number of studies reporting biological sex	84 (81)	75 (82)	2 (100)	7 (64)
Percent of females in studies that reported sex (%)	56	55	57	61

Data are n (%)

**Table 2:** Demographic, study design, and main results for the studies that included an assessment of transitioning to diabetes care in youth-onset type 2 diabetes

Author/ Year	Country	PubMed ID	Study Design	Population Demographics	Main Results
**Youth-onset Type 2 Diabetes only**					
Agarwal S. et al. 2018 [92]	USA	29,377,258	SEARCH for Diabetes in Youth Cohort Study Longitudinal follow-up ≥ 1 year after age 18 years	Y-T2D *N* = 182 18–25 years	Higher odds of poor glycemic control (HbA1c > 9%) related to transfer of care (OR 4.5, 95% CI 1.8, 11.2) and no care (OR 4.6, 95% CI 1.4, 14.6)
Rasmussen B. et al. 2016 [91]	Australia and Denmark	26,037,014	Purposive sample Diabetes Clinic Qualitative semi-structured interview	Early onset T2D *N* = 26 19–42 years	Diabetes management was difficult during transitions associated with guilt, feelings of low self-worth, anxiety, and depression
**Youth-onset Type 2 Diabetes and Youth-onset Type 1 Diabetes (Both)**					
Sauder K. et al. 2021 [121]	USA	34,376,501	SEARCH for Diabetes in Youth Cohort Study Cross-sectional and Longitudinal	At 8 years post diagnosis Y-T2D, *N* = 230 Y-T1D, *N* = 1,885 At 13 years post diagnosis Y-T2D, *N* = 84 Y-T1D, *N* = 649 11–26 years	Y-T2D: ≥3 HbA1c tests/year associated with specialist care but not related to HbA1c or microvascular complications. Y-T1D: ≥3 HbA1c tests/year associated with lower HbA1c (OR -0.36%, 95% CI -0.65, -0.06), younger age at diagnosis, shorter duration of diabetes, specialist care, and fewer microvascular complications.
Nip A. et al. 2021 [98]	USA	34,675,057	Statewide inpatient database Retrospective analysis of hospital admissions	Y-T2D, *N* = 3304 encounters Y-T1D, *N* = 38,053 encounters 13–25 years	Y-T2D: Diabetes-related hospitalizations unchanged10-11/ 100,000 Y-T1D: Diabetes-related hospitalizations increased from 70/ 100,000 at age 17 to 132/ 100,000 at age 19 youth Both: hospitalizations among Black youth, public insurance, associated with severe conditions
Gupta, A et al. 2019 [120]	Australia	31,295,788	Retrospective analysis of transition diabetes clinic visits	Y-T2D, *N* = 11 Y-T1D, *N* = 91 17–23 years	Multiple episodes of diabetic ketoacidosis associated with fewer clinic appointments, higher HbA1c. No association with psychiatric co-morbidities (anxiety, depression, eating disorder).
Pundyk K. et al. 2021 [94]	Canada	34,001,461	Prospective, population-based cohort study	Y-T2D, *N* = 196 Y-TID, *N* = 456	Y-T2D: higher rates of hospitalization post-transition and fewer medical visits pre- and post-transition compared to Y-T1D
Zhu L. et al., 2021 [100]	Singapore	32,602,276	Youth diabetes cohort Longitudinal study 2 years post-transition to adult care	Y-T2D, *N* = 33 Y-T1D, *N* = 98 70% Chinese, 14% Indian, 15% Malaysian 17–25 years	Both: At transition, 30% have anxiety and 9% depression, no difference between groups At 2 years, 14% and 5% had persistent anxiety and depression, no difference between groups
Raymond J. et al. 2013 [93]	USA	24,416,076	Cross-sectional study Diabetes clinic anonymous questionnaires	*N* = 123 patients and parents Y-T2D: *N* = 7 Y-T1D: *N* = 116 11–19 years	Only 25% of families had discussed a transition care and 10% established a plan. Youth and parents preferred transition discussions occur at 17–18 years of age.
Duke D. et al. 2013 [95]	USA	N/A	Cross-sectional study Tertiary diabetes center	*N* = 40 Y-T2D: *N* = 2 Y-T1D: *N* = 38 19–27 years	Youth who transferred to adult care within the last year reported anxiety about the transition process. 23% of youth had a lapse in care of greater than1 year.
**Unspecified Youth-onset Diabetes**					
Richards J. et al. 2021 [101]	USA	32,578,506	Cross-sectional study Summer camp for youth with diabetes, spina bifida, cerebral palsy, and sickle cell anemia	*N* = 165 youth 6–17 years	Youth who completed chores had higher health care transition readiness scores and better communication with providers compared to youth who did not complete chores.
Wysocki T. et al. 1992 [97]	USA	1,613,115	Cross-sectional study	*N* = 81 young adults with insulin-dependent diabetes 18–22 years	Adjustment to diabetes care in earlier adolescence was a robust predictor of measures of treatment adherence and health care use. A history of major life events predicted poor glycemic control and microalbuminuria.
Ames J. et al. 2021 [99]	USA	32,583,679	Retrospective medical database analysis	Diabetes: *N* = 2156 Autism Spectrum Disorder: *N* = 4123 14–25 years	Heath care utilization among transition-age youth was higher in youth with diabetes compared to youth with autism spectrum disorder
Gerber B. et al. 2007 [96]	USA	17,316,099	Pilot feasibility intervention program	*N* = 19	Internet program with frequent nightly contact was feasible for providing information and addressing social needs in transition-age youth with diabetes

### Transition-related factors characterized by socio-ecological domains of influence

The number of studies categorized by socio-ecological factor collected in Y-T2D and Y-T1D is illustrated in Fig. 2A and reported in Supplemental Table 1.

#### Societal factors

Most studies (82%) analyzed the association of transition care with social determinants of health (Fig. 2A). Specific factors detailed in the studies differed by population, and there was no universal societal factor collected across all studies. In Y-T1D studies, social determinants of health reported included: access to healthcare [23, 26–51], economic stability [23, 25, 30, 47, 52–68], education and policies [23, 25, 26, 30, 32, 35, 37, 40, 42, 44, 46–48, 50, 51, 53, 55–60, 62–65, 68–79], neighborhood and built environment [28, 34, 39, 42, 48, 53, 55, 56, 58–60, 62–64, 69, 70, 74, 79–82], social and community support [23, 25–30, 35, 38, 41, 43, 44, 47, 49, 52–55, 63–68, 70, 71, 73, 74, 77–90], food insecurity [63], and poverty [23, 32, 39, 54, 63]. No studies in Y-T1D investigated structural bias. In Y-T2D/Both studies, two thirds reported on access to healthcare [91–99], structural bias [91], poverty [98], education and policy [94, 96], economic stability [91, 94, 96, 98], social and community support [91, 93, 95, 97, 100, 101], and neighborhood and built environment [91, 94, 98]. No articles reported on the impact of food insecurity on transitioning to adult diabetes care in Y-T2D.

### Community/ relationship factors

#### Health cultural

Reporting of health cultural factors was common and collected in 68% of all studies (Fig. 2). Health cultural factors commonly collected in Y-T1D included: attitudes towards health care [26, 27, 30, 34–37, 39, 45, 47, 51, 55, 56, 59, 61, 63, 68, 72, 73, 77, 79, 82, 83, 87, 102–106], health cultural attitudes [26, 28, 30, 32, 34–36, 41, 43, 46, 47, 55, 56, 58, 59, 63–65, 68, 73, 81–83, 86, 89, 102, 104–109], community or provider diabetes care practices [26, 30, 31, 35, 36, 38, 43, 46, 48, 49, 51, 61, 64, 72, 77, 79, 82–84, 86, 89, 104–106, 108, 110], and multi-disciplinary clinics [39, 45, 64, 73, 83, 86, 104, 106, 108, 110]. Eleven studies reported on at least one health cultural factor in Y-T2D/ Both; attitudes towards health care [91, 93–95, 101], health cultural attitudes [91, 93–95, 97, 100], community or provider diabetes care practices [93, 95, 97], and multi-disciplinary clinics [93, 95–97]. The presence of multi-disciplinary clinic and easy access to adult healthcare provider facilitated transitioning to adult-centered care [91].

#### Psychological

Data documenting mood symptoms, anxiety or depressive disorders were reported in 59% of all studies (Fig. 2A). In Y-T1D, one study examined the impact of early childhood adversity [84], one on post-traumatic stress disorder [31], and multiple assessed disordered eating behaviors [28, 31, 32, 40, 109–113], depressive symptoms [24, 25, 27, 28, 31, 32, 36, 38, 40, 44, 58, 61, 62, 69, 81, 110–115], and anxiety-related symptoms [24, 28, 31, 32, 40, 44, 61, 81, 112, 113, 116]. In Y-T2D, few studies assessed mood symptoms (depression and anxiety [91, 94, 100]) and stress [97]. We did not find any studies that collected data on eating disorders, early childhood adverse events, or post-traumatic stress disorder in Y-T2D.

#### Behavioral

Eighty-four studies (Fig. 2) collected data on behavioral factors. In Y-T1D, studies included data on self-management and medication adherence support [24, 26–29, 31, 33–36, 38, 41–43, 46, 47, 52, 53, 56, 57, 59, 62–70, 72–74, 76, 78–81, 83–87, 90, 102, 105–109, 111, 116–119]. Specific behaviors that impeded outcomes included substance abuse, neglect, use of technologies, transition readiness, and medication dosing. Diabetes self-management strategies were reported on a range of activities, including dietary choices, physical activity, blood glucose monitoring, and medication management. Nine Y-T2D/ Both studies reported on behavioral factors including information on scheduling and attending appointments, self-management strategies, blood glucose monitoring, medication management, and goal setting [91, 93, 95–97, 100, 101, 120, 121]. Additional behaviors that were found to impede outcomes were social isolation [91], risky behaviors (e.g., substance use, previous incarceration) [120], and lack of chore engagement [101]. We did not find any studies that reported on dietary choices and physical activity in Y-T2D during transition.

### Individual/ biological factors

Metabolic biomarkers (body mass index, HBA1c, weight, fructosamine, blood pressure, cholesterol, urine albumin, serum bicarbonate, and ketones) were collected in 58 studies (58% Y-T1D, 50% Y-T2D, 36% of both). No studies examined fasting glucose or fasting insulin concentrations.

### Facilitators and barriers to transitioning care in Y-T2D [96]

Figure 2B illustrates cited barriers experienced in Y-T2D/ Both across the socio-ecological domains. Table 2 details the main results for the studies that included Y-T2D by diagnosis grouping. Most studies evaluated the outcomes of transitioning to adult care while only 2 studies assessed the effect of formulated transition programs [101, 122]. The association of transitioning to adult care was inconsistently related to worsening glycemic control and poor outcomes. For example, the transitioning period was related to 4-fold higher odds of HbA1c greater than 9% in Y-T2D in the SEARCH for Diabetes in Youth Cohort [92]. Though, the transition period was not related to increased diabetes-related hospitalizations in the United States [98]. In contrast, a robust prospective cohort study in Canada described higher rates of hospitalizations and fewer medical visits in the transitioning period in Y-T2D compared to Y-T1D [94]. A retrospective analysis from Australia found poor diabetes-related outcomes related to fewer medical visits [120]. Frequent HbA1c testing in Y-T2D was not a predictor of glycemic outcomes or microvascular complications compared to Y-T1D [123].

Feelings of anxiety, depression, and low self-worth were prevalent among transitioning Y-T2D and Y-T1D, although there was a small sample size of Y-T2D in these studies [91, 95, 100]. Y-T2D reported economic challenges, life transition, stigma, a sense of guilt, and fear of being judged by others as impediments to transitioning to adult-centered care [91]. Other factors related to poor diabetes outcomes in Y-T2D were structural bias, poverty, economic instability, access to quality healthcare, neighborhood and built environment, and social and community support [91, 94, 96, 98].

Four studies investigated the factors contributing to resiliency and mitigating strategies Y-T2D/ Both [93, 94, 96, 101]. Routine performance of household chores [101], easy access to clinics located in close proximity to the pediatric clinic, purposeful developmental age-appropriate diabetes education, older age during transfer to adult care [93], an internet-based program [96], the presence of a multi-disciplinary clinic, and easy access to adult healthcare providers [91] were identified facilitators of transition care. Difficulty establishing rapport with patients, abrupt transition to a new clinic, long waiting period for appointments, and relocation to the new environment were barriers to transitioning to adult care [94, 96]. The presence of a multi-disciplinary clinic and easy access to adult healthcare providers facilitated transitioning to adult-centered care [91].

## Discussion

This scoping review provided a global assessment of socio-ecological factors associated with transitioning to adult-centered diabetes care in Y-T2D compared to Y-T1D. The review confirmed that the transitioning period from pediatric to adult diabetes care is associated with difficulties in diabetes self-management and social barriers to care for both Y-T2D and Y-T1D. Our scoping review supports the American Diabetes Association (ADA) position statement of transitioning care (2011) and the 2025 ADA Standards of Care recommending effective systems-based and translatable transition processes for all youth with diabetes [4, 124]. However, only 8% of studies examined socio-ecological factors in Y-T2D only group, and the evidence quality in Y-T2D studies was predominantly from observational studies with no rigorous randomized studies evaluating transitioning strategies or programs in Y-T2D.

This comprehensive scoping review was the first step towards rigorously mapping the complex transitioning literature on social, economic, psychological, cultural, behavioral, and biological domains of influence. Significant knowledge gaps in understanding the broad range of socio-ecological factors affecting the transition to adult-centered care remain. Based on this scoping review, reporting on the effects of social determinants of health and behavioral self-management factors was common in both Y-T1D and Y-T2D, but fewer studies investigated community and relationship factors (Supplemental Table 1). Fewer than half of studies in Y-T2D reported on psychological factors, which contrasts with data in Y-T1D. Therefore, additional studies are needed to examine transition-related outcomes and transition strategies, especially in psychological domains, that can support the development of effective transitioning programs in Y-T2D.

Understanding the unique barriers and facilitators of transitioning to adult diabetes care in Y-T2D compared to Y-T1D is critical because the disease burdens, complications, correlates, and risk factors markedly differ between the two conditions [125]. In Y-T2D, diabetes-related complications are present at diagnosis in up to 25% of youth, and rates of complications continue to rise, especially during the emerging adult years [126, 127]. Since the period between adolescence and young adulthood is marked by physiological, socio-emotional, and behavioral alterations such as experimentation, risky behaviors, mood changes, feelings of vulnerability, and invincibility [128, 129], designing effective transition programs should include individualized, dynamic, and iterative programs that can accommodate the changing needs of adolescents with Y-T2D [9, 130]. This review showcased poor diabetes-related outcomes in both Y-T1D and Y-T2D. Y-T2D had greater than 3-fold higher odds of worsening glycemia and lack of insurance [92]. Higher rates of diabetes hospitalization and HbA1c were observed in Y-T2D [92, 94, 120], though not in all studies [98]. Disparate results among studies could be due to differences in the natural history of Y-T1D and Y-T2D and/or confounded by the small number of research studies evaluating a limited number of Y-T2D.

No experimental studies examined causative factors associated with poor transition outcomes in Y-T2D. Review of the limited observational studies in the literature suggested that socio-ecological factors across all domains may exacerbate the burdens among Y-T2D who are disproportionately impacted from underserved communities [6]. Social barriers to independence in diabetes self-care may include poverty, restricted access to diabetes-related medications and technologies, structural bias, food insecurity, and difficulty navigating public health insurance [15, 131]. However, a systematic analysis was not possible because the study methodology and transitioning programs were varied in scope, by country and by institution. Inequalities in Y-T2D disease burden may be magnified during the transition from pediatric to adult care, but more data are needed to quantify the factors mediating these disparities.

Despite these study limitations, we identified a range of facilitators and barriers that should be considered when designing diabetes transition modules for Y-T2D. Mood-related symptoms were prominently reported across studies. Anxiety-related symptoms punctuated the transition period and sometimes co-existed with other feelings of stigmatization and worry about socio-economic burdens. Recent analysis in emerging adults from the TODAY iCount ancillary study support the importance of mindfulness and health cultural impacts in Y-T2D, although these studies were not included in this scoping review because they did not include a transition care focus. Diabetes self-efficacy, beliefs about medication, diabetes distress, and social factors were key determinants of medication adherence and glycemic control in Y-T2D [132–134]. Additional studies are also needed to characterize the scope of psychological influences on emerging adults with Y-T2D, especially since we did not identify any articles describing the impact of adverse childhood experiences or other comorbidities (including eating disorders) on health outcomes during the transition period.

Attitudes towards healthcare, the availability of community or private diabetes care practices, and multi-disciplinary clinics also influenced engagement in Y-T2D. Though programmatic data is limited, programs that recognized cultural influences on health behaviors and healthcare utilization (providing telehealth or programs that encouraged early and open dialogue with healthcare providers and adolescents) were feasible and successful in Y-T2D [93, 94, 96, 101]. Intentional efforts to promote transparency and knowledge dissemination to adolescents and emerging adults could improve healthcare access, enhance patient-provider communication, and promote culturally competent diabetes self-management strategies among diverse populations, but requires further investigation. This is an active area of ongoing research, and two studies have recently been published examining health care utilization in Y-T1D [135] and illness experiences in Y-T2D during the transitioning period [136]. Sadness, ambivalence, and fear of the adult environment were consistently reported as important barriers to transitioning to adult care [136].

### Limitations

Studies in Y-T2D were limited because of their relatively small sample size, low power, and diverse study designs that precluded systematic comparison between the two groups. The broad types of studies assessed and few data on transitioning in Y-T2D precluded a comprehensive literature appraisal to determine the direction of associations or extraction of effect size estimates. Finally, to increase analytical rigor, we designed this study to include published peer-reviewed articles only. However, we acknowledge that our scoping review was limited because quality assessments of study designs were not performed, and excluding non-peer-reviewed literature increased the risk of selection bias.

## Conclusion

Transitioning to adult-centered diabetes care is associated with multiple challenges across the socio-ecological domains in Y-T2D, but there is insufficient data to support robust design of Y-T2D transitioning programs. The scope of psychological and behavioral factors that influence transitioning to adult care in Y-T2D is poorly defined. Social determinants of health, the presence of social stigma, mood-related symptoms, and concerns about healthcare system were reported barriers to care in Y-T2D. Additional research is needed to identify culturally sensitive and structural factors to guide health policy and transition programs for successful healthcare continuity in Y-T2D. Targeted interventions that support systems navigation, health culture relationships, reduce social stigma, and address mental-health symptoms are needed to achieve successful transitioning to adult care.

## Electronic supplementary material

Below is the link to the electronic supplementary material.

Supplementary Material 1

## Data Availability

The datasets generated and/or analyzed during the current study are not publicly available but are available from the corresponding author on reasonable request. All articles included in this review are available in the supplementary table.
