# Supplementary material for: Transitioning to adult care in youth-onset diabetes: a scoping review of socio-ecological factors in youth-onset type 2 diabetes compared to type 1 diabetes
Source: BMC Public Health. 2025 May 15;25:1784. doi: 10.1186/s12889-025-22956-1 (PMC12080029; doi:10.1186/s12889-025-22956-1)
Supplement: Supplementary file 1 — Supplementary Material 1 [file 12889_2025_22956_MOESM1_ESM.docx]

**Transitioning to adult care in youth-onset diabetes: A scoping review of socio-ecological factors in youth-onset type 2 diabetes compared to type 1 diabetes.**

**Supplemental Material**

Assumpta O. Ude*^1^, Sydney A. Dixon*^2^, Sophia Glaros^2^, Sue-Ann Arboine^1^, Nancy L. Terry^3^, Tomás Cabeza De Baca^4^, Stephanie T. Chung^2^
*Co-first authors

^1^Clinical Center Nursing Department, National Institutes of Health
^2^Section on Pediatric Diabetes, Obesity, and Metabolism, National Institute of Diabetes & Digestive & Kidney Diseases, National Institutes of Health
^3^National Institutes of Health Library

^4^Obesity and Diabetes Clinical Research Section, Phoenix Epidemiology and Clinical Research Branch, National Institute of Diabetes and Digestive and Kidney Diseases, Phoenix, Arizona,

**Corresponding Author:**

Stephanie T. Chung

[stephanie.chung@nih.gov](mailto:stephanie.chung@nih.gov)

**SUPPLEMENTAL TABLE 1: Demographic characteristics and socio-ecological domains of influence reported in studies included in this scoping review.**

| **Ref** | **First Author** | | | **Pubmed ID** | **Study Demographics** | | | **Socio-ecological factor collected (Yes/No)** | | | | | **Sample size** |
| --- | --- | --- | --- | --- | --- | --- | --- | --- | --- | --- | --- | --- | --- |
|  | **Last name** | **name** | |  | **Diagnosis** | **Language** | **Country** | **SDOH** | **Health Cultural** | **Psychological** | **Behavioral/ attitude** | **Biological** | **Number** |
| [1] | Addala | Ananta | | 31392807 | T1D | English | USA | Yes | Yes | Yes | Yes | Yes | 17 |
| [2] | Castensøe-Seidenfaden | Pernille | | 28099760 | T1D | English | Denmark | Yes | Yes | Yes | Yes | Yes | 126 |
| [3] | Mello | Daniel | | 32353651 | T1D | English | USA | Yes | Yes | No | Yes | Yes | 236 |
| [4] | Brady | Patrick | | 28760008 | T1D | English | USA | Yes | Yes | Yes | Yes | No | 4 |
| [5] | Pyatak | Elizabeth | | 21834462 | T1D | English | USA | Yes | Yes | Yes | Yes | No | 8 |
| [6] | Butalia | Sonia | | 32782975 | T1D | English | Canada | Yes | Yes | No | Yes | No | 7 |
| [7] | Michaud | S. | | 29577410 | T1D | English | Canada | Yes | No | No | No | No | 53 |
| [8] | Abdoli | Samereh | | 28340547 | T1D | English | USA | Yes | No | No | Yes | No | 9 |
| [9] | Alwadiy | Falsai | | 34176613 | T1D | English | Canada | Yes | No | Yes | Yes | Yes | 74 |
| [10] | Karlsson | Agenta | | 17046768 | T1D | English | Sweden | Yes | No | No | Yes | Yes | 32 |
| [11] | Burns | Kharis | | 29034986 | T1D | English | Australia | No | No | No | No | Yes | 39 |
| [12] | Kellet | J | | 29687498 | T1D | English | UK | Yes | No | No | Yes | Yes | 584 |
| [13] | Kime | Nicky | | none | T1D | English | UK | Yes | No | No | Yes | No | 250 |
| [14] | McDowell | Megan E | | 32697881 | T1D | English | USA | Yes | Yes | Yes | Yes | No | 25 |
| [15] | Garvey | Katharine C | | 23807526 | T1D | English | USA | Yes | Yes | No | Yes | Yes | 258 |
| [16] | Gutierrez-Colina | Ana M | | 32848347 | T1D | English | USA | Yes | Yes | Yes | Yes | Yes | 44 |
| [17] | Pyatak | Elizabeth | | 27889401 | T1D | English | USA | No | No | Yes | No | Yes | 75 |
| [18] | Spaic | Tamara | | 31010873 | T1D | English | Canada | Yes | Yes | Yes | Yes | Yes | 205 |
| [19] | Egan | Eileen A | | 25916493 | T1D | English | USA | Yes | Yes | Yes | Yes | Yes | 29 |
| [20] | LaBarbera | Brenton | | 30971099 | T1D | English | USA | Yes | Yes | Yes | Yes | Yes | 177 |
| [21] | Bronner | Madelon B | | 32398086 | T1D | English | Netherlands | Yes | Yes | Yes | Yes | No | 165 |
| [22] | Pyatak | Elizabeth | | 24798586 | T1D | English | USA | Yes | Yes | Yes | Yes | 1 | 20 |
| [23] | Vallis | Michael | | 29171084 | T1D | English | UK | Yes | Yes | Yes | Yes | No | 8596 |
| [24] | While | Alison | | 27678488 | T1D | English | Ireland | Yes | No | Yes | Yes | No | 217 |
| [25] | Markowitz | Jessica | | 22150392 | T1D | English | USA | No | No | Yes | Yes | Yes | 15 |
| [26] | Pasquini | Silvia | | 34657317 | T1D | English | Italy | No | No | Yes | Yes | Yes | 222 |
| [27] | Visagie | Elné | | none | T1D | English | South Africa | Yes | Yes | No | Yes | Yes | 8 |
| [28] | Tsevat | Rebecca K. | | 36067922 | T1D | English | USA | Yes | Yes | No | Yes | No | 138 |
| [29] | Holtz | Bree E. | | none | T1D | English | USA | Yes | Yes | Yes | Yes | No | 12 |
| [30] | Sequeira | Paola | | 25906787 | T1D | English | USA | No | Yes | Yes | Yes | Yes | 81 |
| [31] | Sritharan | Aarooran | | 34886392 | T1D | English | Australia | No | No | Yes | Yes | Yes | 220 |
| [32] | Skedgell | Kyleigh | | 33034097 | T1D | English | USA | Yes | Yes | Yes | Yes | Yes | 62 |
| [33] | Lotstein | Debra | | 23530167 | T1D | English | USA | Yes | Yes | No | No | Yes | 185 |
| [34] | Hilliard | Marisa | | 24089544 | T1D | English | USA | Yes | Yes | No | Yes | No | 12 |
| [35] | Schmidt | Silke | | 29340756 | T1D | English | Germany | Yes | No | No | Yes | No | 285 |
| [36] | Nakhla | Meranda | | 19933731 | T1D | English | Canada | Yes | Yes | No | No | No | 1507 |
| [37] | Pyatak | Elizabeth | | 23935361 | T1D | English | USA | Yes | Yes | No | Yes | No | 8 |
| [38] | Liu | Fang | | 32879087 | T1D | Chinese | China | Yes | No | Yes | Yes | Yes | 342 |
| [39] | Quinn | Sheila | | 2729501 | T1D | English | USA | Yes | No | Yes | No | Yes | 43 |
| [40] | Sullivan-Bolyai | Susan | | 24470041 | T1D | English | USA | No | No | No | No | Yes | 10 |
| [41] | Gee | Leslie | | 17553021 | T1D | English | USA | Yes | Yes | Yes | Yes | Yes | 23 |
| [42] | Wiley | Janice | | 24345040 | T1D | English | Australia | Yes | Yes | No | No | Yes | 150 |
| [43] | Price | Christine | | 22007985 | T1D | English | UK | Yes | Yes | No | No | No | 11 |
| [44] | Hu | Tina | | 35249249 | T1D | English | USA | Yes | Yes | Yes | Yes | Yes | 237 |
| [45] | Perry | Lin | | 22672458 | T1D | English | Australia | Yes | Yes | No | No | No | 26 |
| [46] | Vanelli | Maurizio | | 16295053 | T1D | English | Italy | No | Yes | No | Yes | Yes | 73 |
| [47] | Weyhreter |  | none | | T1D | German | Germany | Yes | No | Yes | Yes | Yes | 60 |
| [48] | Wdowik | Melissa | | 9355372 | T1D | English | USA | Yes | No | Yes | Yes | No | 25 |
| [49] | Grigorian | Ernest | | 35668888 | T1D | English | USA | Yes | No | Yes | Yes | No | 33 |
| [50] | Walsh | Orla M | | 30520613 | T1D | English | Ireland | No | Yes | No | Yes | No | 20 |
| [51] | Flor | Mercè | | 36017376 | T1D | English | Spain | No | Yes | Yes | Yes | No | 11 |
| [52] | VanWalleghem | Norma | | 17306065 | T1D | English | Canada | No | Yes | No | No | No | 373 |
| [53] | Tremblay | Elise S. | | 33223771 | T1D | English | USA | No | Yes | No | No | No | 14 |
| [54] | Alassaf | Abeer | | 28177592 | T1D | English | Jordan | Yes | Yes | No | Yes | Yes | 102 |
| [55] | Lašaitė | Lina | | 27613444 | T1D | English | Lithuania | Yes | Yes | Yes | Yes | Yes | 538 |
| [56] | Berg | Cynthia A. | | 30131398 | T1D | English | USA | Yes | Yes | Yes | Yes | Yes | 247 |
| [57] | Lee Tracy | Eunjin | | 31588160 | T1D | English | USA | Yes | No | Yes | Yes | Yes | 247 |
| [58] | Gray | S. | | 29852520 | T1D | English | UK | Yes | Yes | Yes | No | Yes | 150 |
| [59] | Sawyer | Bailee | | 35615101 | T1D | English | USA | Yes | Yes | Yes | Yes | No | 21 |
| [60] | Berg | Cynthia | | 31095317 | T1D | English | USA | Yes | No | Yes | Yes | Yes | 228 |
| [61] | Leung | Joseph M.W.S. | | 33107064 | T1D | English | Canada | Yes | Yes | Yes | Yes | No | 22 |
| [62] | Simms | MaryJane | | 29456906 | T1D | English | USA | Yes | Yes | No | Yes | Yes | 20 |
| [63] | Perry | Lin | | 20955120 | T1D | English | Australia | Yes | Yes | No | No | Yes | 239 |
| [64] | Giménez | M | | 18644079 | T1D | English | Spain | No | Yes | Yes | Yes | Yes | 74 |
| [65] | Wilson | Valerie | | 21294499 | T1D | English | USA | Yes | Yes | Yes | Yes | No | 23 |
| [66] | Hegelson | Vicki | | 25294781 | T1D | English | USA | No | No | Yes | Yes | Yes | 240 |
| [67] | Hanna | Kathleen | | 22017460 | T1D | English | USA | No | No | Yes | Yes | Yes | 113 |
| [68] | Sparud-Lundin | Carina | | 20423440 | T1D | English | Sweden | Yes | No | Yes | No | No | 13 |
| [69] | Johnson | Barbara | | 23952498 | T1D | English | UK | Yes | Yes | Yes | Yes | Yes | 96 |
| [70] | Ersig | Anne | | 26831378 | T1D | English | USA | Yes | Yes | No | Yes | No | 15 |
| [71] | Halldórsdóttir | Hildur | | 19996464 | T1D | Icelandic | Iceland | Yes | Yes | Yes | Yes | Yes | 56 |
| [72] | Lorenzo | Montali | | 34561133 | T1D | English | Italy | Yes | Yes | Yes | Yes | No | 22 |
| [73] | Markowitz | Benjamin | | 31298715 | T1D | English | Canada | Yes | No | Yes | Yes | Yes | 33 |
| [74] | Helgeson | Vicki | | 23157171 | T1D | English | USA | Yes | Yes | Yes | Yes | Yes | 118 |
| [75] | Weigensberg | Marc | | 29552422 | T1D | English | USA | Yes | Yes | Yes | No | Yes | 37 |
| [76] | Chiang | Yueh-Tao | | 32292126 | T1D | English | China | No | No | Yes | Yes | Yes | 14 |
| [77] | Fisher | Eldad | | 30116747 | T1D | English | Israel | Yes | No | No | No | No | 261 |
| [78] | Ng | Ashley | | 34544625 | T1D | English | Australia | Yes | Yes | No | Yes | No | 46 |
| [79] | Williams | Sarah | | 32307818 | T1D | English | Canada | Yes | Yes | No | Yes | No | 93 |
| [80] | Insabella | Glendessa | | 17659065 | T1D | English | USA | Yes | No | Yes | Yes | Yes | 117 |
| [81] | Sattoe | Jane | | 34969691 | T1D | English | Netherlands | Yes | Yes | No | Yes | No | 164 |
| [82] | Baechle | Christina | | 35104568 | T1D | English | Germany | Yes | Yes | Yes | Yes | Yes | 487 |
| [83] | Baucom | Katherine J W | | 30024228 | T1D | English | USA | Yes | No | Yes | Yes | Yes | 197 |
| [84] | Little | Jeanne | | 27692972 | T1D | English | USA | Yes | Yes | Yes | Yes | Yes | 33 |
| [85] | Ali | Naushad | | none | T1D | English | Australia | Yes | Yes | No | Yes | Yes | 356 |
| [86] | Beal | Sarah | | 27345693 | T1D | English | USA | No | No | No | Yes | No | 163 |
| [87] | Colver | Allan | | 30032726 | T1D | English | UK | Yes | Yes | Yes | Yes | No | 150 |
| [88] | Suchy | Yana | | 31822304 | T1D | English | USA | Yes | Yes | Yes | Yes | Yes | 247 |
| [89] | Helgeson | Vicki S. | | 33566266 | T1D | English | USA | Yes | No | Yes | Yes | Yes | 88 |
| [90] | Agarwal | Shivani | | 29377258 | T2D | English | USA | Yes | Yes | No | Yes | Yes | 182 |
| [91] | Rasmussen | Bodil | | 26037014 | T2D | English | Australia | Yes | Yes | Yes | Yes | No | 26 |
| [92] | Raymond | Jennifer | | 24416076 | Both/Unspecified | English | USA | Yes | Yes | No | Yes | No | 123 |
| [93] | Duke | Danny C | | none | Both/Unspecified | English | USA | Yes | Yes | No | Yes | No | 40 |
| [94] | Richards | Jordan | | 32578506 | Both/Unspecified | English | USA | Yes | Yes | Yes | Yes | No | 165 |
| [95] | Sauder | Katherine | | 34376501 | Both/Unspecified | English | USA | Yes | No | No | Yes | Yes | 230 |
| [96] | Nip | Angel Siu Ying | | 34675057 | Both/Unspecified | English | USA | Yes | No | No | No | No | # |
| [97] | Gupta | Akhil | | 31295788 | Both/Unspecified | English | Australia | No | Yes | Yes | Yes | Yes | 102 |
| [98] | Wysocki | Tim | | 1613115 | Both/Unspecified | English | USA | Yes | Yes | Yes | Yes | Yes | 81 |
| [99] | Pundyk | Katherine | | 34001461 | Both/Unspecified | English | Canada | Yes | Yes | Yes | No | No | 652 |
| [100] | Zhu | Ling | | 32602276 | Both/Unspecified | English | Singapore | Yes | Yes | Yes | Yes | Yes | 131 |
| [101] | Ames | Jennifer | | 32583679 | Both/Unspecified | English | USA | Yes | Yes | No | No | No | 47509 |
| [102] | Gerber | Ben | | 17316099 | Both/Unspecified | English | USA | Yes | Yes | No | Yes | No | 19 |
| [103] | Pacaud | Daniele | |  | T1D | English | Canada | Yes | Yes | No | Yes | No | 154 |
| [104] | Goethals | Eveline R | | 32597383 | T1D | English | USA | No | Yes | Yes | Yes | No | 531 |

# 34,749 admission encounters for youth-onset type 1 diabetes and 3,304 for youth-onset type 2 diabetes were analyzed. T1D: youth-onset type 1 diabetes, T2D: youth-onset type 2 diabetes

REFERENCES

1. Addala A, Igudesman D, Kahkoska AR, Muntis FR, Souris KJ, Whitaker KJ, Pratley RE, Mayer-Davis E: **The interplay of type 1 diabetes and weight management: A qualitative study exploring thematic progression from adolescence to young adulthood**. *Pediatric diabetes* 2019, **20**(7):974-985.

2. Castensøe-Seidenfaden P, Jensen AK, Smedegaard H, Hommel E, Husted GR, Pedersen-Bjergaard U, Teilmann G: **Clinical, behavioural and social indicators for poor glycaemic control around the time of transfer to adult care: a longitudinal study of 126 young people with diabetes**. *Diabet Med* 2017, **34**(5):667-675.

3. Mello D, Wiebe D, Baker AC, Butner J, Berg C: **Neighborhood disadvantage, parent-adolescent relationship quality, and type 1 diabetes in late adolescents transitioning to early emerging adulthood**. *Soc Sci Med* 2020, **255**:113010.

4. Brady PJ, Song HJ, Butler J, 3rd: **Using an Expert Panel to Develop Social Support Program Sequencing for Young Adults With Type 1 Diabetes**. *Health Promot Pract* 2017, **18**(6):789-797.

5. Pyatak E: **Participation in occupation and diabetes self-management in emerging adulthood**. *Am J Occup Ther* 2011, **65**(4):462-469.

6. Butalia S, McGuire KA, Dyjur D, Mercer J, Pacaud D: **Youth with diabetes and their parents' perspectives on transition care from pediatric to adult diabetes care services: A qualitative study**. *Health Sci Rep* 2020, **3**(3):e181.

7. Michaud S, Dasgupta K, Bell L, Yale JF, Anjachak N, Wafa S, Nakhla M: **Adult care providers' perspectives on the transition to adult care for emerging adults with Type 1 diabetes: a cross-sectional survey**. *Diabetic medicine : a journal of the British Diabetic Association* 2018, **35**(7):846-854.

8. Abdoli S, Hardy LR, Hall J: **The Complexities of "Struggling to Live Life"**. *The Diabetes educator* 2017, **43**(2):206-215.

9. Alwadiy F, Mok E, Dasgupta K, Rahme E, Frei J, Nakhla M: **Association of Self-Efficacy, Transition Readiness and Diabetes Distress With Glycemic Control in Adolescents With Type 1 Diabetes Preparing to Transition to Adult Care**. *Can J Diabetes* 2021, **45**(5):490-495.

10. Karlsson A, Arman M, Wikblad K: **Teenagers with type 1 diabetes--a phenomenological study of the transition towards autonomy in self-management**. *Int J Nurs Stud* 2008, **45**(4):562-570.

11. Burns K, Farrell K, Myszka R, Park K, Holmes-Walker DJ: **Access to a youth-specific service for young adults with type 1 diabetes mellitus is associated with decreased hospital length of stay for diabetic ketoacidosis**. *Intern Med J* 2018, **48**(4):396-402.

12. Kellett J, Sampson M, Swords F, Murphy HR, Clark A, Howe A, Price C, Datta V, Myint KS: **Young people's experiences of managing Type 1 diabetes at university: a national study of UK university students**. *Diabet Med* 2018, **35**(8):1063-1071.

13. Kime N: **Join us on our journey": Exploring the experiences of children and young people with type 1 diabetes and their parents**. *Practical Diabetes* 2014, **31**(1):24-28.

14. McDowell ME, Litchman ML, Guo JW: **The transition experiences of adolescents with type 1 diabetes from paediatric to adult care providers**. *Child: care, health and development* 2020, **46**(6):692-702.

15. Garvey KC, Wolpert HA, Laffel LM, Rhodes ET, Wolfsdorf JI, Finkelstein JA: **Health care transition in young adults with type 1 diabetes: barriers to timely establishment of adult diabetes care**. *Endocrine practice : official journal of the American College of Endocrinology and the American Association of Clinical Endocrinologists* 2013, **19**(6):946-952.

16. Gutierrez-Colina AM, Corathers S, Beal S, Baugh H, Nause K, Kichler JC: **Young Adults With Type 1 Diabetes Preparing to Transition to Adult Care: Psychosocial Functioning and Associations With Self-Management and Health Outcomes**. *Diabetes Spectr* 2020, **33**(3):255-263.

17. Pyatak EA, Sequeira PA, Vigen CL, Weigensberg MJ, Wood JR, Montoya L, Ruelas V, Peters AL: **Clinical and Psychosocial Outcomes of a Structured Transition Program Among Young Adults With Type 1 Diabetes**. *The Journal of adolescent health : official publication of the Society for Adolescent Medicine* 2017, **60**(2):212-218.

18. Spaic T, Robinson T, Goldbloom E, Gallego P, Hramiak I, Lawson ML, Malcolm J, Mahon J, Morrison D, Parikh A *et al*: **Closing the Gap: Results of the Multicenter Canadian Randomized Controlled Trial of Structured Transition in Young Adults With Type 1 Diabetes**. *Diabetes care* 2019, **42**(6):1018-1026.

19. Egan EA, Corrigan J, Shurpin K: **Building the bridge from pediatric to adult diabetes care: making the connection**. *The Diabetes educator* 2015, **41**(4):432-443.

20. LaBarbera B, Dvorak J, Zhang Y, Jeter K, Talsania M, Beck J: **Diabetes-related events in adolescents and young adults: opportunities for psychosocial interventions**. *Psychology, health & medicine* 2019, **24**(9):1148-1157.

21. Bronner MB, Peeters MAC, Sattoe JNT, van Staa A: **The impact of type 1 diabetes on young adults' health-related quality of life**. *Health Qual Life Outcomes* 2020, **18**(1):137.

22. Pyatak EA, Sequeira PA, Whittemore R, Vigen CP, Peters AL, Weigensberg MJ: **Challenges contributing to disrupted transition from paediatric to adult diabetes care in young adults with type 1 diabetes**. *Diabetic medicine : a journal of the British Diabetic Association* 2014, **31**(12):1615-1624.

23. Vallis M, Willaing I, Holt RIG: **Emerging adulthood and Type 1 diabetes: insights from the DAWN2 Study**. *Diabet Med* 2018, **35**(2):203-213.

24. While AE, Heery E, Sheehan AM, Coyne I: **Health-related quality of life of young people with long-term illnesses before and after transfer from child to adult healthcare**. *Child: care, health and development* 2017, **43**(1):144-151.

25. Markowitz JT, Laffel LM: **Transitions in care: support group for young adults with Type 1 diabetes**. *Diabet Med* 2012, **29**(4):522-525.

26. Pasquini S, Rinaldi E, Da Prato G, Csermely A, Indelicato L, Zaffani S, Santi L, Sabbion A, Maffeis C, Bonora E, Trombetta M: **Growing up with type 1 diabetes mellitus: Data from the Verona Diabetes Transition Project**. *Diabetic medicine : a journal of the British Diabetic Association* 2022, **39**(4):e14719.

27. Visagie E, E. van Rensburg, and E. Deacon.: **Social support effects on diabetes management by South African emerging adults: A replication and extension study.** *Taylor & Francis*

2018:504-509.

28. Tsevat RK, Weitzman ER, Wisk LE: **Indicators of Healthcare Transition Progress Among College Youth With Type 1 Diabetes**. *Acad Pediatr* 2023, **23**(4):737-746.

29. Holtz BE MK, Holmstrom AJ, Cotten SR, Hershey DD, Dunneback JK, Vega JJ, Wood MA: **Teen and parental perspectives regarding transition of care in type 1 diabetes**. *Children and Youth Services Review* 2020, **110**(104800).

30. Sequeira PA, Pyatak EA, Weigensberg MJ, Vigen CP, Wood JR, Ruelas V, Montoya L, Cohen M, Speer H, Clark S, Peters AL: **Let's Empower and Prepare (LEAP): Evaluation of a Structured Transition Program for Young Adults With Type 1 Diabetes**. *Diabetes care* 2015, **38**(8):1412-1419.

31. Sritharan A, Osuagwu UL, Ratnaweera M, Simmons D: **Eight-Year Retrospective Study of Young Adults in a Diabetes Transition Clinic**. *Int J Environ Res Public Health* 2021, **18**(23).

32. Skedgell KK, Cao VT, Gallagher KA, Anderson BJ, Hilliard ME: **Defining features of diabetes resilience in emerging adults with type 1 diabetes**. *Pediatric diabetes* 2021, **22**(2):345-353.

33. Lotstein DS, Seid M, Klingensmith G, Case D, Lawrence JM, Pihoker C, Dabelea D, Mayer-Davis EJ, Gilliam LK, Corathers S *et al*: **Transition from pediatric to adult care for youth diagnosed with type 1 diabetes in adolescence**. *Pediatrics* 2013, **131**(4):e1062-1070.

34. Hilliard ME, Perlus JG, Clark LM, Haynie DL, Plotnick LP, Guttmann-Bauman I, Iannotti RJ: **Perspectives from before and after the pediatric to adult care transition: a mixed-methods study in type 1 diabetes**. *Diabetes care* 2014, **37**(2):346-354.

35. Schmidt S, Markwart H, Bomba F, Muehlan H, Findeisen A, Kohl M, Menrath I, Thyen U: **Differential effect of a patient-education transition intervention in adolescents with IBD vs. diabetes**. *Eur J Pediatr* 2018, **177**(4):497-505.

36. Nakhla M, Daneman D, To T, Paradis G, Guttmann A: **Transition to adult care for youths with diabetes mellitus: findings from a Universal Health Care System**. *Pediatrics* 2009, **124**(6):e1134-1141.

37. Pyatak EA, Florindez D, Weigensberg MJ: **Adherence decision making in the everyday lives of emerging adults with type 1 diabetes**. *Patient preference and adherence* 2013, **7**:709-718.

38. Liu F, Li L, Xu R, Li X, Xie Y, Zhang H: **Status of social avoidance and distress in emerging adults with Type 1 diabetes mellitus and its association with self-management and glycemic control**. *Zhong Nan Da Xue Xue Bao Yi Xue Ban* 2020, **45**(7):834-839.

39. Quinn SM, Ambrosino JM, Doyle EA, Weyman K, Tamborlane WV, Jastreboff AM: **UTILITY OF PSYCHOLOGICAL SCREENING OF YOUNG ADULTS WITH TYPE 1 DIABETES TRANSITIONING TO ADULT PROVIDERS**. *Endocrine practice : official journal of the American College of Endocrinology and the American Association of Clinical Endocrinologists* 2016, **22**(9):1104-1110.

40. Sullivan-Bolyai S, Bova C, Johnson K, Cullen K, Jaffarian C, Quinn D, Aroke EN, Crawford S, Lee MM, Gupta O: **Engaging teens and parents in collaborative practice: perspectives on diabetes self-management**. *The Diabetes educator* 2014, **40**(2):178-190.

41. Gee L, Smith TL, Solomon M, Quinn MT, Lipton RB: **The clinical, psychosocial, and socioeconomic concerns of urban youth living with diabetes**. *Public Health Nurs* 2007, **24**(4):318-328.

42. Wiley J, Westbrook M, Long J, Greenfield JR, Day RO, Braithwaite J: **Multidisciplinary diabetes team care: the experiences of young adults with Type 1 diabetes**. *Health Expect* 2015, **18**(5):1783-1796.

43. Price CS, Corbett S, Lewis-Barned N, Morgan J, Oliver LE, Dovey-Pearce G: **Implementing a transition pathway in diabetes: a qualitative study of the experiences and suggestions of young people with diabetes**. *Child: care, health and development* 2011, **37**(6):852-860.

44. Hu TY, Price J, Pierce JS, Gannon AW: **The association between pediatric mental health disorders and type 1 diabetes-related outcomes**. *Pediatric diabetes* 2022, **23**(4):507-515.

45. Perry L, Lowe JM, Steinbeck KS, Dunbabin JS: **Services doing the best they can: service experiences of young adults with type 1 diabetes mellitus in rural Australia**. *Journal of clinical nursing* 2012, **21**(13-14):1955-1963.

46. Vanelli M, Caronna S, Adinolfi B, Chiari G, Gugliotta M, Arsenio L: **Effectiveness of an uninterrupted procedure to transfer adolescents with Type 1 diabetes from the Paediatric to the Adult Clinic held in the same hospital: eight-year experience with the Parma protocol**. *Diabetes, nutrition & metabolism* 2004, **17**(5):304-308.

47. Weyhreter H HR: **What becomes of children with diabetes mellitus in adulthood?** *Diab Stoffw* 1996, **5**:56-62.

48. Wdowik MJ, Kendall PA, Harris MA: **College students with diabetes: using focus groups and interviews to determine psychosocial issues and barriers to control**. *The Diabetes educator* 1997, **23**(5):558-562.

49. Grigorian EG, Litchman ML, Porter ME, Blanchette JE, Allen NA: **Financial Barriers in Emerging Adults With Type 1 Diabetes: A Qualitative Analysis**. *Diabetes Spectr* 2022, **35**(2):190-197.

50. Walsh Ó, Wynne M, M OD, O’Hara MC, Geoghegan R: **The Perceptions of Patients, their Parents and Healthcare Providers on the Transition of Young Adults with Type 1 Diabetes to Adult Services in the West of Ireland**. *Irish medical journal* 2018, **111**(7):787.

51. Vidal Flor M, Jansà IMM, Yoldi Vergara C, Cardona-Hernández R, Giménez Alvárez M, Conget Donlo I, Isla Pera P: **Type 1 Diabetes Patient Experiences Before and After Transfer from a Paediatric to an Adult Hospital**. *Patient preference and adherence* 2022, **16**:2229-2246.

52. Van Walleghem N, MacDonald CA, Dean HJ: **Building connections for young adults with type 1 diabetes mellitus in Manitoba: feasibility and acceptability of a transition initiative**. *Chronic Dis Can* 2006, **27**(3):130-134.

53. Tremblay ES, Ruiz J, Buccigrosso T, Dean T, Garvey K: **Health Care Transition in Youth With Type 1 Diabetes and an A1C >9%: Qualitative Analysis of Pre-Transition Perspectives**. *Diabetes Spectr* 2020, **33**(4):331-338.

54. Alassaf A, Gharaibeh L, Grant C, Punthakee Z: **Predictors of type 1 diabetes mellitus outcomes in young adults after transition from pediatric care**. *J Diabetes* 2017, **9**(12):1058-1064.

55. Lašaitė L, Dobrovolskienė R, Danytė E, Stankutė I, Ražanskaitė-Virbickienė D, Schwitzgebel V, Marčiulionytė D, Verkauskienė R: **Diabetes distress in males and females with type 1 diabetes in adolescence and emerging adulthood**. *Journal of diabetes and its complications* 2016, **30**(8):1500-1505.

56. Berg CA, Wiebe DJ, Suchy Y, Turner SL, Butner J, Munion A, Lansing AH, White PC, Murray M: **Executive Function Predicting Longitudinal Change in Type 1 Diabetes Management During the Transition to Emerging Adulthood**. *Diabetes care* 2018, **41**(11):2281-2288.

57. Tracy EL, Berg CA, Baker AC, Mello D, Litchman ML, Wiebe DJ: **Health-risk Behaviors and Type 1 Diabetes Outcomes in the Transition from Late Adolescence to Early Emerging Adulthood**. *Child Health Care* 2019, **48**(3):285-300.

58. Gray S, Cheetham T, McConachie H, Mann KD, Parr JR, Pearce MS, Colver A: **A longitudinal, observational study examining the relationships of patient satisfaction with services and mental well-being to their clinical course in young people with Type 1 diabetes mellitus during transition from child to adult health services**. *Diabetic medicine : a journal of the British Diabetic Association* 2018, **35**(9):1216-1222.

59. Sawyer B, Hilliard E, Hackney KJ, Stastny S: **Barriers and Strategies for Type 1 Diabetes Management Among Emerging Adults: A Qualitative Study**. *Clin Med Insights Endocrinol Diabetes* 2022, **15**:11795514221098389.

60. Berg CA, Wiebe DJ, Lee Tracy E, Kelly CS, Mello D, Turner SL, Butner JE, Munion AK, Mansfield JH, White PC *et al*: **Parental Involvement and Executive Function in Emerging Adults with Type 1 Diabetes**. *Journal of pediatric psychology* 2019, **44**(8):970-979.

61. Leung J, Tang TS, Lim CE, Laffel LM, Amed S: **The four I's of adolescent transition in type 1 diabetes care: A qualitative study**. *Diabetic medicine : a journal of the British Diabetic Association* 2021, **38**(7):e14443.

62. Simms M, Baumann K, Monaghan M: **Health Communication Experiences of Emerging Adults with Type 1 Diabetes**. *Clin Pract Pediatr Psychol* 2017, **5**(4):415-425.

63. Perry L, Steinbeck KS, Dunbabin JS, Lowe JM: **Lost in transition? Access to and uptake of adult health services and outcomes for young people with type 1 diabetes in regional New South Wales**. *The Medical journal of Australia* 2010, **193**(8):444-449.

64. Giménez M, Lara M, Vidal M, Jansà M, Conget I: **Disturbed eating behaviours and glycaemic control in young subjects with Type 1 diabetes transferred from a paediatric to an adult diabetes unit**. *Diabetic medicine : a journal of the British Diabetic Association* 2008, **25**(7):884-885.

65. Wilson V: **Students' experiences of managing type 1 diabetes**. *Paediatr Nurs* 2010, **22**(10):25-28.

66. Helgeson VS, Reynolds KA, Siminerio LM, Becker DJ, Escobar O: **Cognitive adaptation theory as a predictor of adjustment to emerging adulthood for youth with and without type 1 diabetes**. *J Psychosom Res* 2014, **77**(6):484-491.

67. Hanna KM, Weaver MT, Stump TE, Dimeglio LA, Miller AR, Crowder S, Fortenberry JD: **Initial findings: primary diabetes care responsibility among emerging adults with type 1 diabetes post high school and move out of parental home**. *Child: care, health and development* 2013, **39**(1):61-68.

68. Sparud-Lundin C, Ohrn I, Danielson E: **Redefining relationships and identity in young adults with type 1 diabetes**. *Journal of advanced nursing* 2010, **66**(1):128-138.

69. Johnson B, Elliott J, Scott A, Heller S, Eiser C: **Medical and psychological outcomes for young adults with Type 1 diabetes: no improvement despite recent advances in diabetes care**. *Diabet Med* 2014, **31**(2):227-231.

70. Ersig AL, Tsalikian E, Coffey J, Williams JK: **Stressors in Teens with Type 1 Diabetes and Their Parents: Immediate and Long-Term Implications for Transition to Self-Management**. *J Pediatr Nurs* 2016, **31**(4):390-396.

71. Halldórsdóttir H, Steinsdóttir FK, Gudmundsdóttir A, Smári J, Arnarson EO: **[Clinical status and treatment adherence of young adults with type one diabetes mellitus following transition to adult health care]**. *Laeknabladid* 2009, **95**(11):755-761.

72. Montali L, Zulato E, Cornara M, Ausili D, Luciani M: **Barriers and facilitators of type 1 diabetes self-care in adolescents and young adults**. *J Pediatr Nurs* 2022, **62**:136-143.

73. Markowitz B, Pritlove C, Mukerji G, Lavery JV, Parsons JA, Advani A: **The 3i Conceptual Framework for Recognizing Patient Perspectives of Type 1 Diabetes During Emerging Adulthood**. *JAMA Netw Open* 2019, **2**(7):e196944.

74. Helgeson VS, Reynolds KA, Snyder PR, Palladino DK, Becker DJ, Siminerio L, Escobar O: **Characterizing the transition from paediatric to adult care among emerging adults with Type 1 diabetes**. *Diabetic medicine : a journal of the British Diabetic Association* 2013, **30**(5):610-615.

75. Weigensberg MJ, Vigen C, Sequeira P, Spruijt-Metz D, Juarez M, Florindez D, Provisor J, Peters A, Pyatak EA: **Diabetes Empowerment Council: Integrative Pilot Intervention for Transitioning Young Adults With Type 1 Diabetes**. *Glob Adv Health Med* 2018, **7**:2164956118761808.

76. Chiang YT, Yu HY, Lo FS, Chen CW, Huang TT, Chang CW, Moons P: **Emergence of a butterfly: the life experiences of type 1 diabetes Taiwanese patients during the 16-25 years old transition period**. *Int J Qual Stud Health Well-being* 2020, **15**(1):1748362.

77. Fisher E, Lazar L, Shalitin S, Yackobovitch-Gavan M, de Vries L, Oron T, Tenenbaum A, Phillip M, Lebenthal Y: **Association between Glycemic Control and Clinic Attendance in Emerging Adults with Type 1 Diabetes: A Tertiary Center Experience**. *J Diabetes Res* 2018, **2018**:9572817.

78. Ng AH, Pedersen ML, Rasmussen B, Rothmann MJ: **Needs of young adults with type 1 diabetes during life transitions - An Australian-Danish experience**. *Patient education and counseling* 2022, **105**(5):1338-1341.

79. Williams S, Shulman R, Allwood Newhook LA, Power H, Guttmann A, Smith S, Knight J, Chafe R: **A province wide review of transition practices for young adult patients with type 1 diabetes**. *J Eval Clin Pract* 2021, **27**(1):111-118.

80. Insabella G, Grey M, Knafl G, Tamborlane W: **The transition to young adulthood in youth with type 1 diabetes on intensive treatment**. *Pediatric diabetes* 2007, **8**(4):228-234.

81. Sattoe J, Peeters M, Bronner M, van Staa A: **Transfer in care and diabetes distress in young adults with type 1 diabetes mellitus**. *BMJ Open Diabetes Res Care* 2021, **9**(2).

82. Baechle C, Stahl-Pehe A, Castillo K, Lange K, Holl RW, Rosenbauer J: **Course of screening-based depression in young adults with a long type 1 diabetes duration: Prevalence and transition probabilities - A cohort study**. *Diabetes research and clinical practice* 2022, **185**:109220.

83. Baucom KJW, Turner SL, Tracy EL, Berg CA, Wiebe DJ: **Depressive symptoms and diabetes management from late adolescence to emerging adulthood**. *Health psychology : official journal of the Division of Health Psychology, American Psychological Association* 2018, **37**(8):716-724.

84. Little JM, Odiaga JA, Minutti CZ: **Implementation of a Diabetes Transition of Care Program**. *J Pediatr Health Care* 2017, **31**(2):215-221.

85. Ali N, J. Longson, et al.: **Comparison of compliance and outcomes in adolescents with type 1 diabetes mellitus attending a co-located pediatric and transition diabetes service.** *Journal of Transition Medicine* 2021, **3**(1).

86. Beal SJ, Riddle IK, Kichler JC, Duncan A, Houchen A, Casnellie L, Woodward J, Corathers SD: **The Associations of Chronic Condition Type and Individual Characteristics With Transition Readiness**. *Acad Pediatr* 2016, **16**(7):660-667.

87. Colver A, Pearse R, Watson RM, Fay M, Rapley T, Mann KD, Le Couteur A, Parr JR, McConachie H, Transition Collaborative G: **How well do services for young people with long term conditions deliver features proposed to improve transition?** *BMC Health Serv Res* 2018, **18**(1):337.

88. Suchy Y, Butner J, Wiebe DJ, Campbell M, Turner SL, Berg CA: **Executive Cognitive Functions and Behavioral Control Differentially Predict HbA1c in Type 1 Diabetes across Emerging Adulthood**. *J Int Neuropsychol Soc* 2020, **26**(4):353-363.

89. Helgeson VS: **Diabetes burnout among emerging adults with type 1 diabetes: a mixed methods investigation**. *J Behav Med* 2021, **44**(3):368-378.

90. Agarwal S, Raymond JK, Isom S, Lawrence JM, Klingensmith G, Pihoker C, Corathers S, Saydah S, D'Agostino RB, Jr., Dabelea D: **Transfer from paediatric to adult care for young adults with Type 2 diabetes: the SEARCH for Diabetes in Youth Study**. *Diabet Med* 2018, **35**(4):504-512.

91. Rasmussen B, Terkildsen Maindal H, Livingston P, Dunning T, Lorentzen V: **Psychosocial factors impacting on life transitions among young adults with type 2 diabetes: an Australian - Danish qualitative study**. *Scand J Caring Sci* 2016, **30**(2):320-329.

92. Raymond JK, Duke DC, Shimomaeda L, Harris MA: **Looking forward to transition: perspectives on transition from pediatric to adult diabetes care**. *Diabetes Manag (Lond)* 2013, **3**(4).

93. Duke DC RJ, Shimomaeda L, Marris MA: **Recommendations for transition from pediatric to adult diabetes care: patient's perspectives**. *Diabetes management (London)* 2013, **3**(4):297-304.

94. Richards J, Nazareth M, van Tilburg MAL, Jain N, Hart L, Faldowski RA, Coltrane C, Hooper SR, Ferris M, Rak E: **Engagement in Household Chores in Youth With Chronic Conditions: Health care Transition Implications**. *OTJR (Thorofare N J)* 2021, **41**(1):6-14.

95. Sauder KA, Stafford JM, Ehrlich S, Lawrence JM, Liese AD, Marcovina S, Mottl AK, Pihoker C, Saydah S, Shah AS *et al*: **Disparities in Hemoglobin A(1c) Testing During the Transition to Adulthood and Association With Diabetes Outcomes in Youth-Onset Type 1 and Type 2 Diabetes: The SEARCH for Diabetes in Youth Study**. *Diabetes care* 2021.

96. Nip ASY, Lodish M: **Trend of Diabetes-Related Hospital Admissions During the Transition Period From Adolescence to Adulthood in the State of California**. *Diabetes Care* 2021, **44**(12):2723-2728.

97. Gupta A, Taylor F, O'Sullivan T, Simmons D: **Characteristics of young adults with multiple episodes of diabetic ketoacidosis**. *Intern Med J* 2019, **49**(7):911-914.

98. Wysocki T, Hough BS, Ward KM, Green LB: **Diabetes mellitus in the transition to adulthood: adjustment, self-care, and health status**. *J Dev Behav Pediatr* 1992, **13**(3):194-201.

99. Pundyk KJ, Sellers EAC, Kroeker K, Wicklow BA: **Transition of Youth With Type 2 Diabetes: Predictors of Health-Care Utilization After Transition to Adult Care From Population-Based Administrative Data**. *Canadian journal of diabetes* 2021, **45**(5):451-457.

100. Zhu L, Chandran SR, Tan WB, Xin X, Goh SY, Gardner DS: **Persistent Anxiety Is Associated with Higher Glycemia Post-Transition to Adult Services in Asian Young Adults with Diabetes**. *Diabetes Metab J* 2021, **45**(1):67-76.

101. Ames JL, Massolo ML, Davignon MN, Qian Y, Croen LA: **Healthcare service utilization and cost among transition-age youth with autism spectrum disorder and other special healthcare needs**. *Autism* 2021, **25**(3):705-718.

102. Gerber BS, Solomon MC, Shaffer TL, Quinn MT, Lipton RB: **Evaluation of an internet diabetes self-management training program for adolescents and young adults**. *Diabetes technology & therapeutics* 2007, **9**(1):60-67.

103. Pacaud D, J. F. Yale, et al.: **Problems in transition from pediatric care to adult care for individuals with diabetes.** *Canadian journal of diabetes* 2005, **29**(1):13-18.

104. Goethals ER, La Banca RO, Forbes PW, Telo GH, Laffel LM, Garvey KC: **Health Care Transition in Type 1 Diabetes: Perspectives of Diabetes Care and Education Specialists Caring for Young Adults**. *The Diabetes educator* 2020, **46**(3):252-260.
